# 4-(4-Bromo-3-methyl-1*H*-pyrazol-1-yl)-6-(but-3-yn­yloxy)pyrimidine

**DOI:** 10.1107/S1600536809046194

**Published:** 2009-11-07

**Authors:** Lin-Sen Heng, Yong-Hong Li, Xiang-Dong Mei, Jun Ning

**Affiliations:** a College of Bio-information, Chongqing University of Post and Telecommunications, Chongqing 400065, People’s Republic of China; b Key Laboratory of Pesticide Chemistry and Applications, Ministry of Agriculture, Institute of Plant Protection Academy of Agricultural Sciences, Beijing, 100193, People’s Republic of China

## Abstract

There are two mol­ecules in the asymmetric unit of the title compound, C_12_H_11_BrN_4_O. The dihedral angles between the pyrazole and pyrimidine rings are 1.28 (17) and 1.56 (17)° in the two mol­ecules. In one of the mol­ecules, the but-3-yn­yloxy side chain is disordered over two sets of sites in a 0.714 (8):0.286 (8) ratio.

## Related literature

For background information on pyrimidines, see: Regiec *et al.* (2009 ▶).
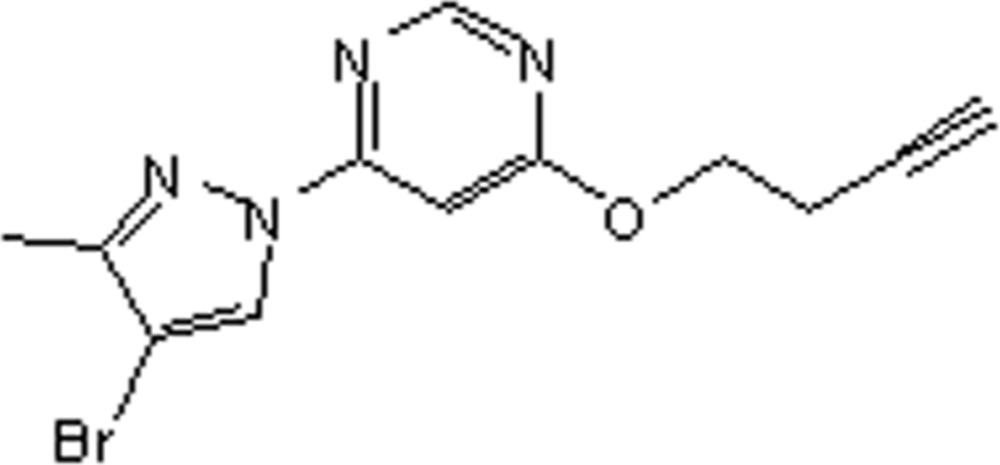


## Experimental

### 

#### Crystal data

C_12_H_11_BrN_4_O
*M* *_r_* = 307.16
Triclinic, 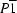

*a* = 7.9053 (16) Å
*b* = 10.045 (2) Å
*c* = 16.807 (3) Å
α = 75.34 (3)°
β = 77.98 (3)°
γ = 80.78 (3)°
*V* = 1254.7 (4) Å^3^
*Z* = 4
Mo *K*α radiation
μ = 3.27 mm^−1^
*T* = 173 K
0.11 × 0.10 × 0.07 mm

#### Data collection

Rigaku Saturn724+ CCD diffractometer
Absorption correction: multi-scan (*CrystalClear*; Rigaku, 2008 ▶) *T*_min_ = 0.715, *T*_max_ = 0.803
15373 measured reflections
5693 independent reflections
4988 reflections with *I* > 2σ(*I*)
*R*_int_ = 0.038

#### Refinement

*R*[*F* ^2^ > 2σ(*F* ^2^)] = 0.047
*wR*(*F* ^2^) = 0.106
*S* = 1.15
5693 reflections
344 parameters
48 restraints
H-atom parameters constrained
Δρ_max_ = 0.38 e Å^−3^
Δρ_min_ = −0.40 e Å^−3^

### 

Data collection: *CrystalClear* (Rigaku, 2008 ▶); cell refinement: *CrystalClear*; data reduction: *CrystalClear*; program(s) used to solve structure: *SHELXS97* (Sheldrick, 2008 ▶); program(s) used to refine structure: *SHELXL97* (Sheldrick, 2008 ▶); molecular graphics: *SHELXTL* (Sheldrick, 2008 ▶); software used to prepare material for publication: *SHELXL97*.

## Supplementary Material

Crystal structure: contains datablocks I, global. DOI: 10.1107/S1600536809046194/hb5193sup1.cif

Structure factors: contains datablocks I. DOI: 10.1107/S1600536809046194/hb5193Isup2.hkl

Additional supplementary materials:  crystallographic information; 3D view; checkCIF report

## Figures and Tables

**Fig. 1.**
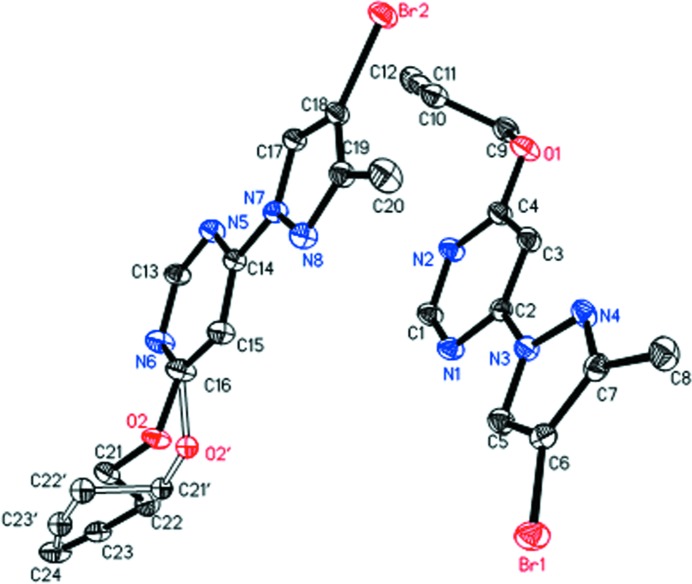
The molecular structure of the title compound showing 30% probability displacement ellipsoids.

## supplementary crystallographic information

### Experimental

The title compound (0.1 g) was dissolved in the mixed solvent of ethanol and acetone (20 ml) at room temperature. Colourless blocks of (I) were obtained through slow evaporation after two weeks.

### Refinement

All the hydrogen atoms were placed at their geometrical position with C—H = 0.93–0.98Å and *U*_iso_ (H) = 1.2–1.5 *U*_ep_ (C).

### Crystal data

**Table:**

C_12_H_11_BrN_4_O	*Z* = 4
*M**_r_* = 307.16	*F*(000) = 616
Triclinic, *P*1	*D*_x_ = 1.626 Mg m^−^^3^
Hall symbol: -P 1	Mo *K*α radiation, λ = 0.71073 Å
*a* = 7.9053 (16) Å	Cell parameters from 536 reflections
*b* = 10.045 (2) Å	θ = 2.2–27.5°
*c* = 16.807 (3) Å	µ = 3.27 mm^−^^1^
α = 75.34 (3)°	*T* = 173 K
β = 77.98 (3)°	Plate, colorless
γ = 80.78 (3)°	0.11 × 0.10 × 0.07 mm
*V* = 1254.7 (4) Å^3^	

### Data collection

**Table:**

Rigaku Saturn724+ CCD diffractometer	5693 independent reflections
Radiation source: sealed tube	4988 reflections with *I* > 2σ(*I*)
graphite	*R*_int_ = 0.038
ω scans at fixed χ = 45°	θ_max_ = 27.5°, θ_min_ = 2.7°
Absorption correction: multi-scan (*CrystalClear*; Rigaku, 2008)	*h* = −10→10
*T*_min_ = 0.715, *T*_max_ = 0.803	*k* = −13→13
15373 measured reflections	*l* = −21→21

### Refinement

**Table:**

Refinement on *F*^2^	Primary atom site location: structure-invariant direct methods
Least-squares matrix: full	Secondary atom site location: difference Fourier map
*R*[*F*^2^ > 2σ(*F*^2^)] = 0.047	Hydrogen site location: inferred from neighbouring sites
*wR*(*F*^2^) = 0.106	H-atom parameters constrained
*S* = 1.15	*w* = 1/[σ^2^(*F*_o_^2^) + (0.036*P*)^2^ + 1.1156*P*] where *P* = (*F*_o_^2^ + 2*F*_c_^2^)/3
5693 reflections	(Δ/σ)_max_ = 0.001
344 parameters	Δρ_max_ = 0.38 e Å^−^^3^
48 restraints	Δρ_min_ = −0.40 e Å^−^^3^

### Special details

**Table:**

Geometry. All e.s.d.'s (except the e.s.d. in the dihedral angle between two l.s. planes) are estimated using the full covariance matrix. The cell e.s.d.'s are taken into account individually in the estimation of e.s.d.'s in distances, angles and torsion angles; correlations between e.s.d.'s in cell parameters are only used when they are defined by crystal symmetry. An approximate (isotropic) treatment of cell e.s.d.'s is used for estimating e.s.d.'s involving l.s. planes.
Refinement. Refinement of *F*^2^ against ALL reflections. The weighted *R*-factor *wR* and goodness of fit *S* are based on *F*^2^, conventional *R*-factors *R* are based on *F*, with *F* set to zero for negative *F*^2^. The threshold expression of *F*^2^ > σ(*F*^2^) is used only for calculating *R*-factors(gt) *etc*. and is not relevant to the choice of reflections for refinement. *R*-factors based on *F*^2^ are statistically about twice as large as those based on *F*, and *R*- factors based on ALL data will be even larger.

### Fractional atomic coordinates and isotropic or equivalent isotropic displacement parameters (Å^2^)

**Table:**

	*x*	*y*	*z*	*U*_iso_*/*U*_eq_	Occ. (<1)
Br1	0.84764 (4)	0.85935 (4)	−0.01999 (2)	0.04160 (12)	
Br2	−0.32369 (4)	0.71052 (4)	0.25637 (2)	0.04014 (12)	
O1	−0.0831 (3)	0.4253 (2)	0.12494 (14)	0.0390 (6)	
N1	0.4057 (3)	0.4528 (3)	0.15609 (17)	0.0313 (6)	
N2	0.1464 (3)	0.3392 (3)	0.19584 (17)	0.0329 (6)	
N3	0.4330 (3)	0.6484 (3)	0.04879 (16)	0.0275 (6)	
N4	0.3723 (4)	0.7482 (3)	−0.01432 (16)	0.0319 (6)	
N5	0.0933 (3)	0.3312 (3)	0.46819 (17)	0.0311 (6)	
N6	0.3526 (4)	0.2288 (3)	0.52183 (18)	0.0364 (7)	
N7	0.0905 (3)	0.5264 (3)	0.36020 (15)	0.0267 (5)	
N8	0.1708 (3)	0.6315 (3)	0.30304 (17)	0.0322 (6)	
C1	0.3082 (4)	0.3559 (3)	0.2019 (2)	0.0323 (7)	
H1A	0.3588	0.2898	0.2443	0.039*	
C2	0.3293 (4)	0.5457 (3)	0.09735 (18)	0.0262 (6)	
C3	0.1647 (4)	0.5414 (3)	0.0840 (2)	0.0309 (7)	
H3A	0.1140	0.6078	0.0418	0.037*	
C4	0.0784 (4)	0.4343 (3)	0.1362 (2)	0.0313 (7)	
C5	0.5956 (4)	0.6658 (3)	0.0570 (2)	0.0315 (7)	
H5A	0.6636	0.6096	0.0961	0.038*	
C6	0.6397 (4)	0.7794 (3)	−0.0020 (2)	0.0304 (7)	
C7	0.4990 (4)	0.8284 (3)	−0.04569 (19)	0.0302 (7)	
C8	0.4879 (5)	0.9490 (4)	−0.1191 (2)	0.0400 (8)	
H8A	0.3742	0.9589	−0.1359	0.060*	
H8B	0.5802	0.9331	−0.1657	0.060*	
H8C	0.5020	1.0337	−0.1035	0.060*	
C9	−0.1643 (5)	0.3004 (4)	0.1696 (2)	0.0388 (8)	
H9A	−0.0746	0.2192	0.1722	0.047*	
H9B	−0.2516	0.2868	0.1387	0.047*	
C10	−0.2516 (5)	0.3097 (4)	0.2568 (2)	0.0387 (8)	
H10A	−0.1668	0.3282	0.2874	0.046*	
H10B	−0.3476	0.3863	0.2548	0.046*	
C11	−0.3206 (4)	0.1772 (4)	0.2997 (2)	0.0404 (8)	
C12	−0.3688 (5)	0.0678 (4)	0.3306 (2)	0.0461 (9)	
H12	−0.4077	−0.0205	0.3555	0.055*	
C13	0.1847 (4)	0.2382 (3)	0.5180 (2)	0.0324 (7)	
H13A	0.1221	0.1683	0.5563	0.039*	
C14	0.1859 (4)	0.4271 (3)	0.41388 (19)	0.0259 (6)	
C15	0.3608 (4)	0.4307 (3)	0.4096 (2)	0.0319 (7)	
H15A	0.4244	0.4988	0.3701	0.038*	
C16	0.4367 (4)	0.3271 (4)	0.4674 (2)	0.0384 (8)	
C17	−0.0815 (4)	0.5332 (3)	0.35647 (19)	0.0285 (7)	
H17A	−0.1629	0.4726	0.3900	0.034*	
C18	−0.1110 (4)	0.6466 (3)	0.2937 (2)	0.0294 (7)	
C19	0.0468 (4)	0.7055 (3)	0.2624 (2)	0.0315 (7)	
C20	0.0820 (5)	0.8313 (4)	0.1948 (2)	0.0460 (9)	
H20A	0.2043	0.8464	0.1873	0.069*	
H20B	0.0071	0.9119	0.2101	0.069*	
H20C	0.0578	0.8183	0.1426	0.069*	
O2	0.6027 (4)	0.3390 (4)	0.4735 (3)	0.0316 (10)	0.714 (8)
C21	0.6792 (6)	0.2448 (5)	0.5416 (3)	0.0337 (13)	0.714 (8)
H21A	0.5903	0.2319	0.5931	0.040*	0.714 (8)
H21B	0.7758	0.2861	0.5521	0.040*	0.714 (8)
C22	0.7475 (7)	0.1058 (6)	0.5210 (3)	0.0374 (13)	0.714 (8)
H22A	0.6528	0.0660	0.5078	0.045*	0.714 (8)
H22B	0.8417	0.1173	0.4716	0.045*	0.714 (8)
C23	0.8143 (6)	0.0124 (5)	0.5927 (3)	0.0347 (13)	0.714 (8)
O2'	0.6200 (12)	0.3001 (11)	0.4464 (6)	0.027 (2)*	0.286 (8)
C21'	0.7129 (14)	0.1783 (13)	0.4919 (7)	0.027 (3)*	0.286 (8)
H21C	0.8333	0.1649	0.4611	0.032*	0.286 (8)
H21D	0.6554	0.0960	0.4953	0.032*	0.286 (8)
C22'	0.7188 (17)	0.1897 (14)	0.5783 (9)	0.035 (3)*	0.286 (8)
H22C	0.7883	0.2653	0.5750	0.042*	0.286 (8)
H22D	0.5993	0.2135	0.6070	0.042*	0.286 (8)
C23'	0.7965 (16)	0.0586 (14)	0.6274 (9)	0.034 (3)*	0.286 (8)
C24	0.8639 (5)	−0.0575 (4)	0.6544 (3)	0.0473 (10)	
H24	0.9032	−0.1129	0.7034	0.057*	

### Atomic displacement parameters (Å^2^)

**Table:**

	*U*^11^	*U*^22^	*U*^33^	*U*^12^	*U*^13^	*U*^23^
Br1	0.03268 (19)	0.0426 (2)	0.0462 (2)	−0.01365 (15)	−0.00338 (15)	−0.00121 (16)
Br2	0.03508 (19)	0.0429 (2)	0.0398 (2)	0.00172 (15)	−0.01630 (15)	−0.00052 (15)
O1	0.0290 (12)	0.0405 (14)	0.0411 (14)	−0.0147 (10)	−0.0134 (10)	0.0147 (11)
N1	0.0258 (13)	0.0306 (14)	0.0333 (15)	−0.0030 (11)	−0.0065 (11)	0.0011 (11)
N2	0.0297 (14)	0.0314 (14)	0.0331 (15)	−0.0035 (11)	−0.0099 (11)	0.0041 (11)
N3	0.0273 (13)	0.0274 (13)	0.0259 (13)	−0.0075 (10)	−0.0050 (10)	0.0006 (10)
N4	0.0358 (15)	0.0311 (14)	0.0279 (14)	−0.0059 (11)	−0.0121 (12)	0.0015 (11)
N5	0.0278 (14)	0.0290 (14)	0.0337 (15)	−0.0083 (11)	−0.0055 (11)	0.0011 (11)
N6	0.0294 (14)	0.0332 (15)	0.0392 (16)	−0.0066 (12)	−0.0089 (12)	0.0093 (12)
N7	0.0238 (13)	0.0253 (13)	0.0276 (14)	−0.0041 (10)	−0.0023 (10)	−0.0009 (10)
N8	0.0309 (14)	0.0280 (14)	0.0327 (15)	−0.0074 (11)	−0.0045 (11)	0.0036 (11)
C1	0.0286 (16)	0.0314 (17)	0.0326 (18)	−0.0019 (13)	−0.0088 (13)	0.0021 (13)
C2	0.0248 (15)	0.0282 (16)	0.0232 (15)	−0.0035 (12)	−0.0028 (12)	−0.0021 (12)
C3	0.0335 (17)	0.0287 (16)	0.0284 (17)	−0.0009 (13)	−0.0109 (13)	0.0002 (13)
C4	0.0291 (16)	0.0324 (17)	0.0311 (17)	−0.0017 (13)	−0.0095 (13)	−0.0028 (13)
C5	0.0304 (17)	0.0311 (17)	0.0315 (17)	−0.0043 (13)	−0.0086 (13)	−0.0013 (13)
C6	0.0287 (16)	0.0308 (17)	0.0314 (17)	−0.0035 (13)	−0.0027 (13)	−0.0086 (13)
C7	0.0329 (17)	0.0310 (17)	0.0257 (16)	−0.0076 (13)	−0.0017 (13)	−0.0049 (13)
C8	0.046 (2)	0.0354 (19)	0.0338 (19)	−0.0089 (16)	−0.0081 (16)	0.0040 (15)
C9	0.0338 (18)	0.044 (2)	0.038 (2)	−0.0123 (15)	−0.0103 (15)	−0.0008 (16)
C10	0.0334 (18)	0.0395 (19)	0.043 (2)	−0.0052 (15)	−0.0098 (15)	−0.0055 (16)
C11	0.0293 (17)	0.048 (2)	0.041 (2)	−0.0049 (15)	−0.0144 (15)	0.0006 (17)
C12	0.047 (2)	0.045 (2)	0.042 (2)	−0.0207 (18)	−0.0114 (17)	0.0103 (17)
C13	0.0255 (16)	0.0324 (17)	0.0359 (18)	−0.0082 (13)	−0.0044 (13)	0.0005 (14)
C14	0.0255 (15)	0.0239 (15)	0.0274 (16)	−0.0044 (12)	−0.0049 (12)	−0.0028 (12)
C15	0.0282 (16)	0.0279 (16)	0.0352 (18)	−0.0050 (13)	−0.0066 (13)	0.0027 (13)
C16	0.0284 (17)	0.0383 (19)	0.042 (2)	−0.0063 (14)	−0.0097 (15)	0.0064 (15)
C17	0.0261 (15)	0.0295 (16)	0.0295 (16)	−0.0026 (12)	−0.0064 (13)	−0.0047 (13)
C18	0.0290 (16)	0.0295 (16)	0.0298 (16)	0.0007 (13)	−0.0102 (13)	−0.0055 (13)
C19	0.0324 (17)	0.0306 (17)	0.0295 (17)	−0.0039 (13)	−0.0072 (13)	−0.0014 (13)
C20	0.052 (2)	0.036 (2)	0.045 (2)	−0.0116 (17)	−0.0146 (18)	0.0088 (16)
O2	0.0213 (16)	0.029 (2)	0.040 (2)	−0.0071 (13)	−0.0081 (15)	0.0042 (17)
C21	0.027 (2)	0.037 (3)	0.037 (3)	−0.0047 (19)	−0.011 (2)	−0.003 (2)
C22	0.036 (3)	0.037 (3)	0.035 (3)	−0.002 (2)	−0.006 (2)	−0.003 (2)
C23	0.026 (2)	0.034 (3)	0.040 (3)	−0.0020 (18)	−0.003 (2)	−0.003 (2)
C24	0.0302 (18)	0.051 (2)	0.049 (2)	−0.0037 (16)	−0.0071 (16)	0.0095 (18)

### Geometric parameters (Å, °)

**Table:**

Br1—C6	1.879 (3)	C10—H10A	0.9900
Br2—C18	1.877 (3)	C10—H10B	0.9900
O1—C4	1.349 (4)	C11—C12	1.177 (5)
O1—C9	1.460 (4)	C12—H12	0.9500
N1—C1	1.322 (4)	C13—H13A	0.9500
N1—C2	1.346 (4)	C14—C15	1.375 (4)
N2—C4	1.335 (4)	C15—C16	1.385 (4)
N2—C1	1.344 (4)	C15—H15A	0.9500
N3—C5	1.363 (4)	C16—O2	1.363 (4)
N3—N4	1.371 (3)	C16—O2'	1.417 (10)
N3—C2	1.404 (4)	C17—C18	1.369 (4)
N4—C7	1.324 (4)	C17—H17A	0.9500
N5—C13	1.318 (4)	C18—C19	1.410 (4)
N5—C14	1.341 (4)	C19—C20	1.490 (4)
N6—C16	1.324 (4)	C20—H20A	0.9800
N6—C13	1.330 (4)	C20—H20B	0.9800
N7—C17	1.364 (4)	C20—H20C	0.9800
N7—N8	1.374 (3)	O2—C21	1.458 (6)
N7—C14	1.402 (4)	C21—C22	1.512 (8)
N8—C19	1.329 (4)	C21—H21A	0.9900
C1—H1A	0.9500	C21—H21B	0.9900
C2—C3	1.374 (4)	C22—C23	1.464 (7)
C3—C4	1.379 (4)	C22—H22A	0.9900
C3—H3A	0.9500	C22—H22B	0.9900
C5—C6	1.348 (4)	C23—C24	1.198 (6)
C5—H5A	0.9500	O2'—C21'	1.448 (16)
C6—C7	1.415 (4)	C21'—C22'	1.496 (18)
C7—C8	1.500 (4)	C21'—H21C	0.9900
C8—H8A	0.9800	C21'—H21D	0.9900
C8—H8B	0.9800	C22'—C23'	1.481 (19)
C8—H8C	0.9800	C22'—H22C	0.9900
C9—C10	1.503 (5)	C22'—H22D	0.9900
C9—H9A	0.9900	C23'—C24	1.218 (13)
C9—H9B	0.9900	C24—H24	0.9500
C10—C11	1.474 (5)		
			
C4—O1—C9	118.1 (2)	N5—C14—N7	114.8 (3)
C1—N1—C2	114.5 (3)	C15—C14—N7	121.2 (3)
C4—N2—C1	114.5 (3)	C14—C15—C16	114.6 (3)
C5—N3—N4	112.1 (2)	C14—C15—H15A	122.7
C5—N3—C2	127.4 (3)	C16—C15—H15A	122.7
N4—N3—C2	120.5 (2)	N6—C16—O2	119.1 (3)
C7—N4—N3	104.9 (2)	N6—C16—C15	124.1 (3)
C13—N5—C14	114.2 (3)	O2—C16—C15	116.3 (3)
C16—N6—C13	114.3 (3)	N6—C16—O2'	117.6 (5)
C17—N7—N8	112.7 (2)	O2—C16—O2'	27.1 (3)
C17—N7—C14	127.3 (3)	C15—C16—O2'	114.6 (5)
N8—N7—C14	120.0 (2)	N7—C17—C18	104.8 (3)
C19—N8—N7	104.9 (2)	N7—C17—H17A	127.6
N1—C1—N2	128.1 (3)	C18—C17—H17A	127.6
N1—C1—H1A	116.0	C17—C18—C19	107.6 (3)
N2—C1—H1A	116.0	C17—C18—Br2	125.8 (2)
N1—C2—C3	123.8 (3)	C19—C18—Br2	126.6 (2)
N1—C2—N3	114.4 (3)	N8—C19—C18	110.0 (3)
C3—C2—N3	121.8 (3)	N8—C19—C20	121.6 (3)
C2—C3—C4	115.4 (3)	C18—C19—C20	128.5 (3)
C2—C3—H3A	122.3	C19—C20—H20A	109.5
C4—C3—H3A	122.3	C19—C20—H20B	109.5
N2—C4—O1	118.8 (3)	H20A—C20—H20B	109.5
N2—C4—C3	123.7 (3)	C19—C20—H20C	109.5
O1—C4—C3	117.5 (3)	H20A—C20—H20C	109.5
C6—C5—N3	105.7 (3)	H20B—C20—H20C	109.5
C6—C5—H5A	127.2	C16—O2—C21	118.4 (3)
N3—C5—H5A	127.2	O2—C21—C22	111.2 (5)
C5—C6—C7	107.3 (3)	O2—C21—H21A	109.4
C5—C6—Br1	125.9 (3)	C22—C21—H21A	109.4
C7—C6—Br1	126.8 (2)	O2—C21—H21B	109.4
N4—C7—C6	110.0 (3)	C22—C21—H21B	109.4
N4—C7—C8	122.3 (3)	H21A—C21—H21B	108.0
C6—C7—C8	127.7 (3)	C23—C22—C21	109.4 (5)
C7—C8—H8A	109.5	C23—C22—H22A	109.8
C7—C8—H8B	109.5	C21—C22—H22A	109.8
H8A—C8—H8B	109.5	C23—C22—H22B	109.8
C7—C8—H8C	109.5	C21—C22—H22B	109.8
H8A—C8—H8C	109.5	H22A—C22—H22B	108.2
H8B—C8—H8C	109.5	C24—C23—C22	175.5 (6)
O1—C9—C10	111.5 (3)	C16—O2'—C21'	120.6 (8)
O1—C9—H9A	109.3	O2'—C21'—C22'	112.0 (12)
C10—C9—H9A	109.3	O2'—C21'—H21C	109.2
O1—C9—H9B	109.3	C22'—C21'—H21C	109.2
C10—C9—H9B	109.3	O2'—C21'—H21D	109.2
H9A—C9—H9B	108.0	C22'—C21'—H21D	109.2
C11—C10—C9	108.5 (3)	H21C—C21'—H21D	107.9
C11—C10—H10A	110.0	C23'—C22'—C21'	111.4 (12)
C9—C10—H10A	110.0	C23'—C22'—H22C	109.3
C11—C10—H10B	110.0	C21'—C22'—H22C	109.3
C9—C10—H10B	110.0	C23'—C22'—H22D	109.3
H10A—C10—H10B	108.4	C21'—C22'—H22D	109.3
C12—C11—C10	176.1 (4)	H22C—C22'—H22D	108.0
C11—C12—H12	180.0	C24—C23'—C22'	168.1 (14)
N5—C13—N6	128.8 (3)	C23—C24—C23'	39.2 (6)
N5—C13—H13A	115.6	C23—C24—H24	180.0
N6—C13—H13A	115.6	C23'—C24—H24	140.8
N5—C14—C15	124.0 (3)		
			
C5—N3—N4—C7	0.0 (4)	C17—N7—C14—N5	−1.6 (5)
C2—N3—N4—C7	178.9 (3)	N8—N7—C14—N5	178.6 (3)
C17—N7—N8—C19	−0.2 (4)	C17—N7—C14—C15	178.4 (3)
C14—N7—N8—C19	179.6 (3)	N8—N7—C14—C15	−1.3 (4)
C2—N1—C1—N2	0.8 (5)	N5—C14—C15—C16	−0.9 (5)
C4—N2—C1—N1	−0.5 (5)	N7—C14—C15—C16	179.1 (3)
C1—N1—C2—C3	−0.8 (5)	C13—N6—C16—O2	171.0 (4)
C1—N1—C2—N3	−180.0 (3)	C13—N6—C16—C15	−1.2 (6)
C5—N3—C2—N1	−2.5 (5)	C13—N6—C16—O2'	−158.2 (6)
N4—N3—C2—N1	178.8 (3)	C14—C15—C16—N6	1.8 (6)
C5—N3—C2—C3	178.3 (3)	C14—C15—C16—O2	−170.6 (4)
N4—N3—C2—C3	−0.3 (5)	C14—C15—C16—O2'	159.4 (6)
N1—C2—C3—C4	0.6 (5)	N8—N7—C17—C18	0.7 (4)
N3—C2—C3—C4	179.7 (3)	C14—N7—C17—C18	−179.1 (3)
C1—N2—C4—O1	179.4 (3)	N7—C17—C18—C19	−0.8 (4)
C1—N2—C4—C3	0.2 (5)	N7—C17—C18—Br2	179.0 (2)
C9—O1—C4—N2	−9.4 (5)	N7—N8—C19—C18	−0.3 (4)
C9—O1—C4—C3	169.8 (3)	N7—N8—C19—C20	179.5 (3)
C2—C3—C4—N2	−0.3 (5)	C17—C18—C19—N8	0.8 (4)
C2—C3—C4—O1	−179.5 (3)	Br2—C18—C19—N8	−179.1 (2)
N4—N3—C5—C6	0.2 (4)	C17—C18—C19—C20	−179.0 (3)
C2—N3—C5—C6	−178.5 (3)	Br2—C18—C19—C20	1.1 (5)
N3—C5—C6—C7	−0.3 (4)	N6—C16—O2—C21	−1.8 (7)
N3—C5—C6—Br1	178.1 (2)	C15—C16—O2—C21	171.0 (4)
N3—N4—C7—C6	−0.2 (3)	O2'—C16—O2—C21	−96.0 (11)
N3—N4—C7—C8	177.7 (3)	C16—O2—C21—C22	81.1 (6)
C5—C6—C7—N4	0.4 (4)	O2—C21—C22—C23	−176.9 (4)
Br1—C6—C7—N4	−178.0 (2)	C21—C22—C23—C24	10 (7)
C5—C6—C7—C8	−177.5 (3)	N6—C16—O2'—C21'	−8.8 (12)
Br1—C6—C7—C8	4.1 (5)	O2—C16—O2'—C21'	91.8 (13)
C4—O1—C9—C10	84.4 (4)	C15—C16—O2'—C21'	−168.0 (8)
O1—C9—C10—C11	−176.4 (3)	C16—O2'—C21'—C22'	−71.7 (13)
C9—C10—C11—C12	22 (6)	O2'—C21'—C22'—C23'	173.6 (10)
C14—N5—C13—N6	1.1 (5)	C21'—C22'—C23'—C24	2(6)
C16—N6—C13—N5	−0.3 (6)	C22—C23—C24—C23'	−10 (6)
C13—N5—C14—C15	−0.4 (5)	C22'—C23'—C24—C23	−3(5)
C13—N5—C14—N7	179.7 (3)		
